# A Machine-Learning Prognostic Model for Colorectal Cancer Using a Complement-Related Risk Signature

**DOI:** 10.32604/or.2025.066193

**Published:** 2025-10-22

**Authors:** Jun Li, Kangmin Yu, Zhiyong Chen, Dan Xing, Binshan Zha, Wentao Xie, Huan Ouyang, Changjun Yu

**Affiliations:** 1 Department of Vascular Surgery, The First Affiliated Hospital of Anhui Medical University, Hefei, 230022, China; 2 Department of Medical Record Management, The First Affiliated Hospital of Anhui Medical University, Hefei, 230022, China; 3 Department of Gastrointestinal Surgery, The First Affiliated Hospital of Anhui Medical University, Hefei, 230022, China

**Keywords:** Colorectal cancer, complement response, tumor microenvironment, prognostic model, the cancer genome atlas, complement-related risk signature (CRRS)

## Abstract

**Objectives:**

Colorectal cancer (CRC) remains a major contributor to global cancer mortality, ranking second worldwide for cancer-related deaths in 2022, and is characterized by marked heterogeneity in prognosis and therapeutic response. We sought to construct a machine-learning prognostic model based on a complement-related risk signature (CRRS) and to situate this signature within the CRC immune microenvironment.

**Methods:**

Transcriptomic profiles with matched clinical annotations from TCGA and GEO CRC cohorts were analyzed. Prognostic CRRS genes were screened using Cox proportional hazards modeling alongside machine-learning procedures. A random survival forest (RSF) predictor was trained and externally validated. Comparisons of immune infiltration, mutational burden, pathway enrichment, and drug sensitivity were made between risk groups. The function of FAM84A, a key model gene, was examined in CRC cell lines.

**Results:**

The six-gene CRRS model accurately stratified patients by survival outcomes. Low-risk patients exhibited greater immune cell infiltration and higher predicted response to immunotherapy and chemotherapy, while high-risk patients showed enrichment of complement activation and matrix remodeling pathways. FAM84A was shown to promote CRC cell proliferation, migration, and epithelial–mesenchymal transition.

**Conclusion:**

CRRS is a critical modulator of the CRC immune microenvironment. The developed model enables precise risk prediction and supports individualized therapeutic decisions in CRC.

## 1 Introduction

Colorectal cancer (CRC) is among the most common cancers globally and ranks second in cancer-related deaths [1]. Annually, CRC accounts for roughly 1.9 million new cases, with both incidence and death rates continuing to rise globally [2,3]. CRC development commonly follows the adenoma-carcinoma pathway and is shaped by a combination of genetic, environmental, and lifestyle factors, including alterations in diet and gut microbiota [4,5]. Even with advances in early detection technologies such as colonoscopy and tumor marker assays, the majority of CRC diagnoses occur at late stages, frequently accompanied by metastasis and recurrence [6]. Current treatments for advanced CRC, including surgery, chemotherapy, radiotherapy, and targeted therapy, are limited by modest efficacy and significant side effects. Consequently, there is an urgent need to discover novel biomarkers and to better characterize the tumor microenvironment (TME) and immune landscape to enable more precise prognostic assessment and personalized therapy for CRC.

Crosstalk between immune cells and tumor cells is pivotal for driving tumor growth, dissemination, and escape from immune control [7,8]. However, the immune microenvironment is not solely composed of immune cells. Inflammatory responses, immune checkpoints and the complement system, in the tumor surroundings also play essential roles in tumor immune responses and metastasis. As part of the innate immune system, the complement system primarily participates in immune surveillance by recognizing and eliminating foreign pathogens, damaged cells, and tumor cells [9]. Complement activation not only promotes the chemotaxis and activation of immune cells but also enhances antigen presentation and the persistence of immune responses to cooperate in anti-tumor reactions [10]. Evidence indicates that tumor cells evade immune surveillance and facilitate tumor growth and metastasis by modulating complement activation [11]. There is also a complex interaction between the coagulation system and the immune system. The coagulation cascade can support tumor growth and metastasis by promoting thrombus formation, angiogenesis, and the accumulation of immune cells [12]. Within the TME, coagulation factors may promote malignant cell proliferation, invasion, and immune escape via crosstalk with immune cells [13,14]. Although the complement system and coagulation cascade are recognized as key components of the TME, their precise functions and mechanisms in CRC remain insufficiently defined.

Our objective was to build a CRC prognostic model from complement and coagulation gene programs using TCGA and GEO datasets. A Complement-Related Risk Signature (CRRS) score was established to estimate patient outcomes and therapy response. We then investigated FAM84A, demonstrating its clinical significance, mechanistic role in tumor progression, and interplay with the immune milieu—highlighting therapeutic potential. Collectively, the findings explain variability in immune responses and provide a basis for individualized prognosis and targeted interventions.

## 2 Materials and Methods

### 2.1 Clinical Sample Collection

All human CRC tissue samples were purchased from Shanghai Xinchao Biotechnology Co., Ltd. (Shanghai, China), a company specializing in the collection and processing of clinical specimens. A total of 30 human tissue samples were included in this study (15 cases of adenocarcinoma and 15 cases of adjacent small intestinal mucosa), all in the form of tissue microarrays. The corresponding catalog number and batch number are [Catalog No.: HColA030PG07-1; Batch No.: XT22-001-2], ensuring traceability and reproducibility. All research protocols, informed consent forms, and related documents were approved by the Ethics Committee (ZJBB-FM-071).

### 2.2 Data Collection and Preparation

A total of 624 colorectal cancer (CRC) tissue samples and 51 normal colon tissue samples, along with corresponding clinical and pathological information, were sourced from The Cancer Genome Atlas (TCGA). To provide independent validation, additional datasets were retrieved from the Gene Expression Omnibus (GEO), specifically utilizing the GSE39582 cohort. Raw gene expression data were processed by applying a log_2_(x + 1) transformation to the normalized FPKM values using the “limma” package (3.56.2) in R. This procedure, coupled with subsequent normalization steps, minimized the effects of library size variation and stabilized variance across the expression range, resulting in standardized data distributions suitable for downstream analyses.

### 2.3 Screening Differentially Expressed CRRS Genes

A total of 137 CRRS candidates were curated from GeneCards (https://www.genecards.org/). Groupwise comparisons (tumor vs. normal) were performed in R using “limma”. Transcripts were retained as differentially expressed when they satisfied |log_2_FC| > 1 together with FDR < 0.05. This dual-threshold approach ensured that only transcripts with both robust expression differences and strong statistical confidence were included, thereby enhancing biological relevance and minimizing type I errors due to multiple testing.

### 2.4 Consensus Stratification Using the CRRS Panel

Unsupervised consensus clustering was performed in R using the ConsensusClusterPlus package [15] to identify distinct molecular subtypes among CRC patients based on CRRS gene expression. To maximize clustering robustness, the algorithm was iterated 1000 times. Survival outcomes were compared across the resulting clusters. Principal component analysis was applied to visualize and quantify between-group separation and within-group homogeneity of the risk strata.

### 2.5 Construction of Prognostic Signature Using Machine Learning

To link differentially expressed gene markers with overall survival (OS) in colorectal cancer, we first ran univariable Cox proportional hazards models (*p* < 0.05) to identify prognostic candidates. Model development used the TCGA-COAD cohort, with GSE39582 serving for external validation. Batch effects were mitigated using the sva package (v3.50.0). We then constructed 101 prognostic models spanning a broad set of machine-learning algorithms, implemented as previously detailed [16]. For each model, predictive performance was summarized by the concordance index (C-index), calculated across the cohort. Survival differences between risk strata were evaluated by Kaplan–Meier analysis using “survival” (v3.7.0) and “survminer” (v0.5.0). Discrimination was further assessed via time-dependent ROC analysis with “timeROC” (v0.4.0) to obtain AUCs. Finally, univariable and multivariable Cox regression confirmed the model’s independence, with hazard ratios (HRs) and 95% confidence intervals (CIs) reported.

### 2.6 Analysis of Tumor Microenvironment and Gene Set Variation

To delineate the tumor microenvironment (TME), we applied “ESTIMATE” (v1.0.13) to derive StromalScore, ImmuneScore, and ESTIMATEScore and to infer tumor purity from transcriptomic data. Immune-cell infiltration was quantified using seven deconvolution frameworks—XCELL, CIBERSORT-ABS, TIMER, EPIC, QUANTISEQ, MCPCOUNTER, and CIBERSORT—and their outputs were correlated with the risk score to compare infiltration patterns between low- and high-risk subgroups. Single-sample enrichment was performed with “GSVA” (v1.50.5) to generate ssGSEA immune-signature scores for colorectal cancer samples. Functional annotation of the prognostic genes was performed with Metascape, yielding Gene Ontology (GO) terms and KEGG pathway enrichments [17].

### 2.7 Tumor Mutational Burden (TMB) and Immune Function Assessment

Mutation annotation files retrieved via the “TCGAbiolinks” (2.30.4) package enabled comprehensive mutational profiling of CRC cases. Comparative analyses of mutation landscapes and TMB scores were performed using the “maftools” (2.18.0) package [18]. Copy-number variation profiles were analyzed using the GISTIC 2.0 module on the GenePattern platform [19], which identified significantly amplified or deleted chromosomal regions and compared CNV patterns across chromosomal arms between risk groups. CNV findings were visualized with the “ggplot2” (3.5.1) package, providing a graphical overview of genomic alterations.

### 2.8 Prediction of ICB Efficacy and Chemotherapy Sensitivity

To estimate the likelihood of CRC patients benefiting from immunotherapy or chemotherapy, the Tumor Immune Dysfunction and Exclusion (TIDE) platform was utilized to assess tumor immune evasion and dysfunction [20]. TIDE scores were calculated for each patient, with higher values indicating a greater potential for immune escape under treatment. To assess chemotherapy sensitivity, drug response data from the Genomics of Drug Sensitivity in Cancer (GDSC) database were integrated, and the half-maximal inhibitory concentration (IC_50_) of various compounds was estimated. The R package “pRRophetic” (0.5.0) [21] was used to predict and compare the IC_50_ values between distinct CRC risk groups.

### 2.9 Cell Culture

SW480 (Procell, CL-0223; STR-authenticated, Tianjin, China) and HCT116 (Procell, CL-0096; STR-authenticated) colorectal cancer cell lines were cultured in high-glucose DMEM (Thermo Fisher Scientific, 11965092) supplemented with 10% fetal bovine serum (Gibco, 10099141C; Macquarie Park, NSW, Australia) under aseptic conditions. The vendor verified cell-line identity by short tandem repeat profiling and confirmed mycoplasma-negative status. Cells were maintained at 37°C in a humidified incubator with 5% CO_2_. Once cells reached logarithmic growth, they were passaged using trypsin-EDTA (Gibco, Cat# 25200056) for subsequent assays.

### 2.10 Small Interfering RNA Transfection

Stable knockdown of FAM84A was achieved via transfection with a retroviral short hairpin RNA (shRNA) vector (RNAi-Ready pSIREN-RetroQ, Clontech, Mountain View, CA, USA) carrying a puromycin resistance marker. For FAM84A overexpression, the full-length coding sequence was subcloned into the lentiviral vector pLV-CS2.0 under the control of the EF1α promoter. All transfections were performed using Turbofect reagent (#R0531, Thermo Scientific, Waltham, MA, USA), following the manufacturer’s recommendations. Following selection with puromycin, stably transfected cell lines were established for downstream functional studies.

### 2.11 Reverse Transcription Quantitative Real-Time Polymerase Chain Reaction (RT-qPCR)

Total RNA was extracted with TRIzol™ (Thermo Fisher Scientific, Cat# 15596026). Cell lysates were held at room temperature for 5 min to disrupt nucleoprotein complexes, after which chloroform was added at 0.2 mL per 1 mL TRIzol (Sinopharm, Cat# 10006818, Beijing, China). Tubes were tightly capped, vortexed for 15 s, and incubated at RT for 3 min. Phase separation was achieved by centrifugation at 12,000× *g*, 15 min, 4°C; the upper aqueous phase was transferred to a fresh RNase-free tube. RNA was precipitated by adding isopropanol at 0.5 mL per 1 mL TRIzol (Sigma-Aldrich, Cat# I9516), gently mixing, incubating 10 min at RT, and centrifuging at 12,000× *g*, 10 min, 4°C. The pellet was washed once with 1 mL 75% ethanol (Thermo Fisher Scientific, Cat# AM9932), briefly vortexed, and centrifuged at 7500× *g*, 5 min, 4°C. Residual ethanol was removed, and the pellet was air-dried for 5–10 min before assessing RNA purity and concentration. cDNA was generated by reverse transcription for downstream RT-qPCR analysis [22].

RT-qPCR was performed with GAPDH as the internal control, and FAM84A expression was quantified using the 2^–ΔΔCt^ approach [23]. Primer sequences were: FAM84A-F: 5^′^-CACCCACCTCAACTACAGCG-3^′^; FAM84A-R: 5^′^-TCCTCATCATCCGAGAAGAAGT-3^′^. GAPDH-F: 5^′^-GAAGGTGAAGGTCGGAGTC-3^′^; GAPDH-R: 5^′^-GAAGATGGTGATGGGATTTC-3^′^. Thermal cycling conditions: 95°C for 30 s, then 40 cycles of 95°C for 5 s and 60°C for 30 s. Each sample was run in technical triplicate.

### 2.12 Western Blot

Proteins were isolated from cultured cells with RIPA lysis buffer (Servicebio, G2002-100ML, Wuhan, China) per the manufacturer’s protocol. Total protein was quantified using a bicinchoninic acid assay (Servicebio, G2026-200T, Wuhan, China). For SDS–PAGE, 20–30 μg of protein was combined with 5× loading buffer (Servicebio, G2043-50T, Wuhan, China), heated at 100°C for 5 min, and resolved on 10–12% polyacrylamide gels (Epizyme, PG111, Tianjin, China). Proteins were transferred to PVDF membranes (Millipore, IPVH00010, Burlington, MA, USA) using a semi-dry blotter (Trans-Blot Turbo, Bio-Rad, Hercules, CA, USA) at 300 A for 60 min.

Membranes were blocked with 5% non-fat milk in TBST for 90 min at room temperature, followed by overnight incubation at 4°C with primary antibodies (1:1000): FAM84A (Abcam, ab126938, Cambridge, UK), N-cadherin (Abcam, ab76011), E-cadherin (Abcam, ab40772), c-Myc (Abcam, ab32072), CDK6 (Abcam, ab151247), Bax (Abcam, ab216985), and Bcl-2 (Abcam, ab194583). The next day, blots were washed three times in TBST (Servicebio, G0004-500ML, Wuhan, China) and incubated with HRP-conjugated secondary antibodies (1:5000) for 1 h at room temperature. Membranes were washed again in TBST (three times, 10 min each) and then developed using an ECL chemiluminescent substrate (Servicebio, Cat# G2161-200ML, Wuhan, China). Protein bands were visualized with a digital imaging system. GAPDH served as the internal loading control. Densitometric analysis was conducted using Leica LAS EZ software (v3.4; Leica Microsystems, Wetzlar, Germany).

All Western blot experiments were independently repeated at least three times using biological replicates (n = 3). Representative blots and corresponding quantification from these independent assays are presented.

### 2.13 Immunohistochemistry and Scoring

FFPE colorectal cancer sections (4 µm) were prepared and post-fixed in 10% neutral buffered formalin for 24 h before mounting. Slides were baked at 60°C for 2 h, cooled to room temperature, cleared in a descending xylene series (100%→50%), and rehydrated through graded ethanol (100%, 95%, 85%, 75%, 50%; 5 min each). Heat-mediated antigen retrieval was carried out in 10 mM sodium citrate buffer (pH 6.0) at near-boiling temperature for 15 min. Endogenous peroxidase was quenched using a commercial blocking reagent (ZSGB-BIO, SP KIT-B1) for 20 min. Sections were then incubated with primary antibody overnight at 4°C, followed by the appropriate secondary antibody on the next day. Chromogenic development employed 0.05% DAB for 5 min, and nuclei were counterstained with 1× hematoxylin for 2 min. After dehydration in graded ethanol, slides were coverslipped and examined by bright-field microscopy.

Scoring. FAM84A staining was evaluated semi-quantitatively by combining intensity and extent. Intensity was graded as 0 = none, 1 = light/yellow, 2 = brown, 3 = dark brown. The proportion of positive cells received scores of 0 (0–5%), 1 (6–25%), 2 (26–50%), 3 (51–75%), or 4 (>75%). The overall IHC score was calculated as intensity × proportion per published guidance [24]. Scores ≥ 5 were classified as positive expression; scores ≤4 were considered negative.

### 2.14 EdU Proliferation and Transwell Migration/Invasion Assays

CRC cells were plated in 12-well plates at 5 × 10^4^ cells/well under aseptic conditions and allowed to attach. Cells were then incubated with 50 µmol/L EdU working solution (Servicebio, G1601-100T, Wuhan, China) for 2 h. After treatment, cultures were rinsed with PBS (0.01 M, pH 7.4; Servicebio, G4202), fixed in 4% paraformaldehyde, and processed following the EdU kit instructions. The proliferation index was calculated as (EdU-positive nuclei/total nuclei) × 100%.

For Transwell assays, exponentially growing cells were harvested with 0.25% trypsin-EDTA (Gibco, 25200056; Thermo Fisher Scientific, Waltham, MA, USA) and counted. For the migration assay, 200 µL of cell suspension was loaded into the upper chamber and 500 µL of complete medium into the lower well. After 48 h, cells that had traversed the membrane were fixed with 4% paraformaldehyde, stained with 1% crystal violet, and quantified from five randomly selected non-overlapping fields per insert. For the invasion assay, the insert was pre-coated with 50 µL of diluted Matrigel (serum-free medium: Matrigel = 9:1; Corning, 356237, Corning, NY, USA), and all subsequent steps were identical to those used for migration.

## 3 Statistical Analysis

Statistical analyses were performed using GraphPad Prism 9.0 (GraphPad Inc., La Jolla, CA, USA) and R (v4.2.2). Student’s *t*-tests were applied for two-group comparisons, while one-way ANOVA or chi-square tests were used for multiple-group analyses as appropriate. *p < 0.05; **p < 0.01, ***p < 0.001, ****p < 0.0001.

## 4 Results

### 4.1 Identification of Complement-Related (CR)-DEGs Subgroups in CRC

**Figure 1:**
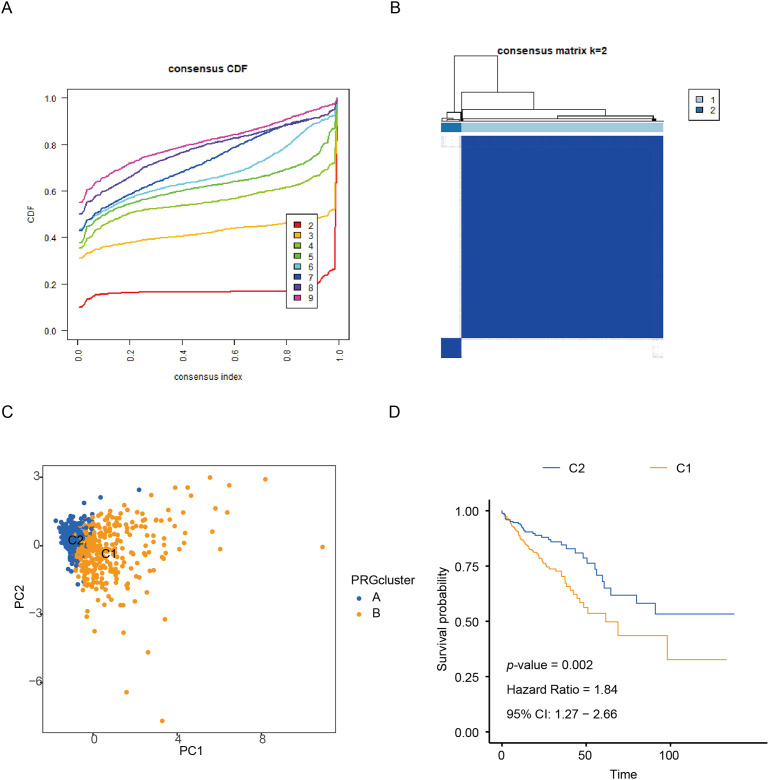
Identification of CR-DEGs Subtypes in COAD. (**A, B**) Unsupervised consensus clustering analysis of COAD; (**C**) PCA analysis of C1 and C2 clusters; (**D**) Kaplan-Meier survival analysis of C1 and C2 clusters

In this study, unsupervised consensus clustering was applied to classify CRC patients based on the expression profiles of six CR-DEGs. Utilizing the optimal cluster number (k = 2), as determined by the algorithm, 624 CRC samples were segregated into two distinct molecular subtypes, designated as C1 and C2. The clustering assignments were further validated through integration with transcriptomic consensus clustering approaches (Fig. 1A,B). PCA effectively separated the samples into the two clusters according to DEGs expression patterns (Fig. 1C). Kaplan-Meier curves showed that patients in cluster C1 had significantly longer overall survival than those in C2 (Fig. 1D).

**Figure 2:**
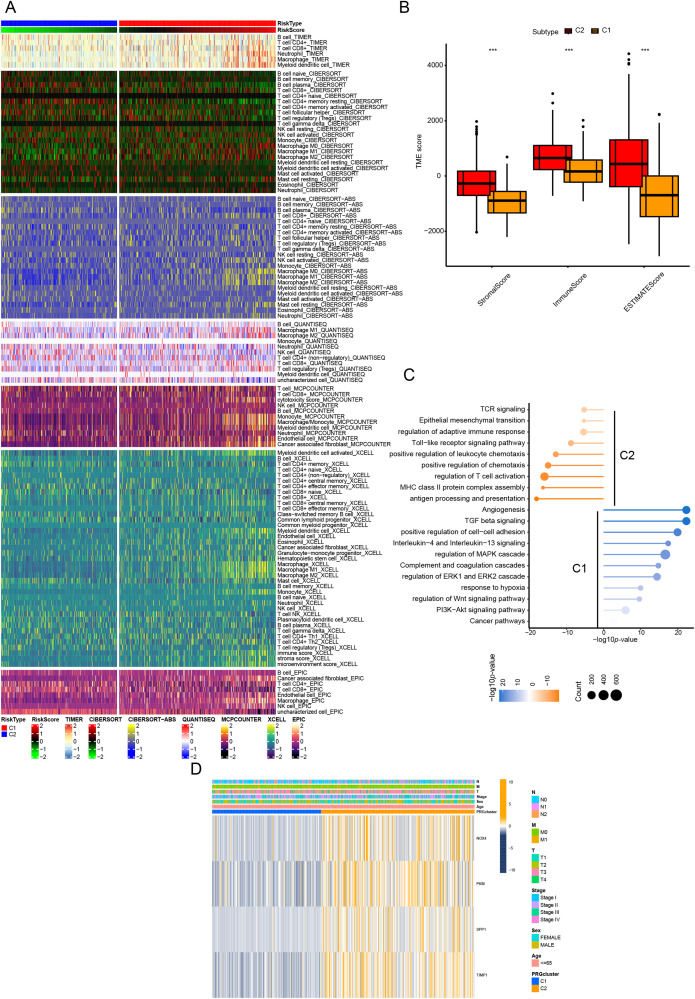
Characteristics of CR-DEGs Subtypes in the TCGA Cohort. (**A**) Tumor immune infiltration analysis of 22 immune cell types in C1 and C2 clusters; (**B**) StromalScore, ImmuneScore, and EstimatedScore between C1 and C2 groups; (**C**) GSVA results of top 20 KEGG pathways in C1 and C2 clusters in TCGA; (**D**) clinical pathological features and expression of CRRG in CRC subtypes. ***p < 0.001

To elucidate the potential mechanisms underlying the prognostic disparities between the two groups, we constructed a comprehensive immune response heatmap by integrating immune infiltration scores calculated from multiple algorithms, including CIBERSORT, ESTIMATE, MCPCOUNTER, ssGSEA, and TIMER (Fig. 2A). Our analysis demonstrated that the C2 subgroup exhibited significantly elevated stromal, immune, and ESTIMATE scores (*p* < 0.001), indicating a more immunologically active and immunogenic tumor microenvironment in these patients (Fig. 2B). To further dissect the functional distinctions between C1 and C2, we conducted enrichment analysis of DEGs, which revealed that C1 was predominantly associated with immune-related pathways, whereas C2 showed significant enrichment in pathways driving malignant tumor progression (Fig. 2C). Moreover, the heatmap visualization also included clinical pathological features and the expression patterns of the six CRR-DEGs, with the majority of these DEGs concentrated within the C1 gene cluster (Fig. 2D).

### 4.2 Construction of the CRRG Prognostic Model

**Figure 3:**
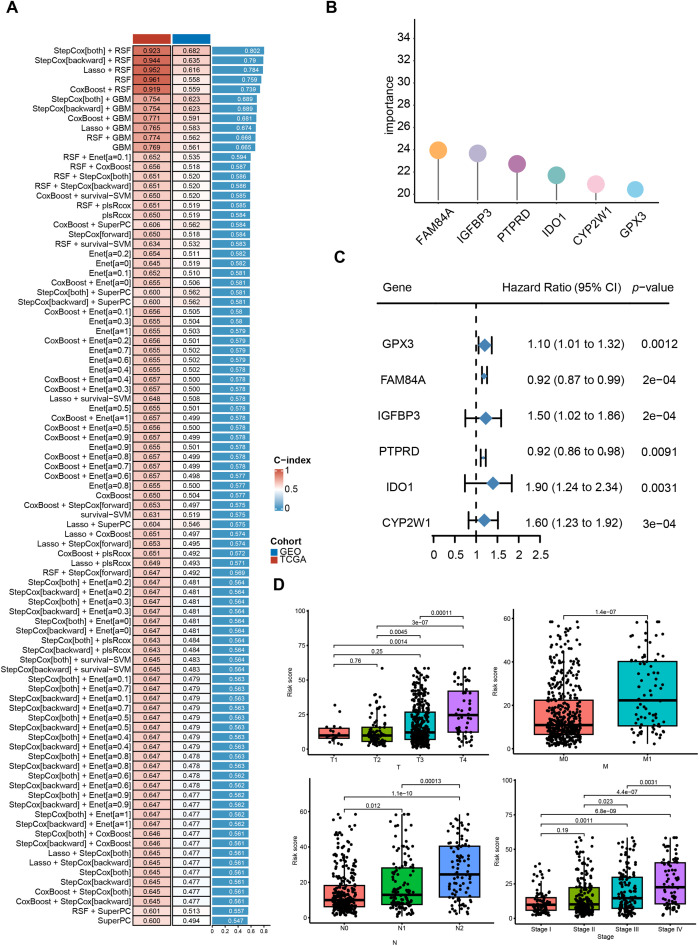
Identification of differentially expressed CRR-DEGs and construction of prognostic risk model, and comprehensive machine learning prognostic model. (**A**) model C-indices in TCGA-CRC and GSE39582, ranked by validation-set mean; (**B**) Importance of six candidate genes shown as a forest plot; (**C**) univariable Cox results for hub genes in the training set; (**D**) correlation analysis between clinical indicators in the TCGA-CRC database

We first performed univariate Cox regression to identify 13 CRRGs significantly correlated with OS, which were subsequently used for prognostic modeling. In the training set, a total of 101 different combinations of feature selection and machine learning algorithms were employed to establish consensus models. For each model, the C-index was calculated to evaluate predictive accuracy across the cohort (Fig. 3A). All models underwent rigorous cross-validation within the training cohort and were further assessed using the independent validation cohort. Among the tested models, the RSF approach achieved the highest mean C-index (Fig. 3B,C), and was therefore selected as the optimal prognostic model. We then examined clinical correlates of the risk score and found that the model-defined high- and low-risk strata differed significantly in T stage, N stage, and overall clinical stage. Exhibited significant differences in TNM staging and overall clinical stage (Fig. 3D). Collectively, these results indicate that the CRRG-based risk signature provides clinically relevant prognostic value for colorectal cancer patients.

### 4.3 Evaluation of the CRRG Model

**Figure 4:**
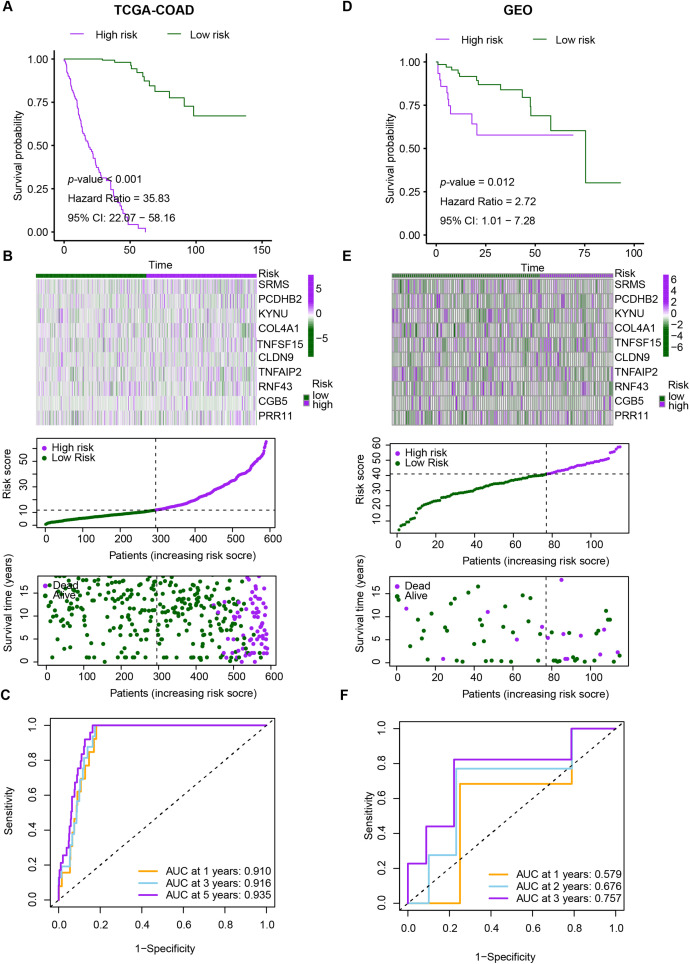
Performance evaluation of CRRG. (**A–C**) Kaplan–Meier curves, ROC analyses, and risk score distributions in the TCGA-CRC cohort. (**D–F**) Corresponding KM, ROC, and risk distribution plots in the GSE29621 cohort

We evaluated the prognostic utility of the CRRG model by partitioning patients into low- and high-risk groups using a data-driven cut-point for the composite score. Kaplan-Meier analyses demonstrated consistently inferior overall survival in the high-risk stratum across the discovery TCGA-CRC and validation GEO cohorts (Fig. 4A). Time-dependent ROC analysis in TCGA-CRC yielded AUCs of 0.910, 0.916, and 0.935 at 1, 3, and 5 years, respectively (Fig. 4B), indicating strong discriminative power. Rank-ordered risk plots with survival overlays further showed a concentration of deaths in the high-risk group (Fig. 4C). These findings were reproduced in the test cohort, supporting the robustness of the CRRG signature. Moreover, both the risk classification and signature gene expression correlated with T stage, N stage, and overall clinical stage, linking the score to disease advancement and potential clinical utility (Fig. 4D–F).

### 4.4 Comprehensive Analysis of Immune Status in High-Risk and Low-Risk CRRG Groups

**Figure 5:**
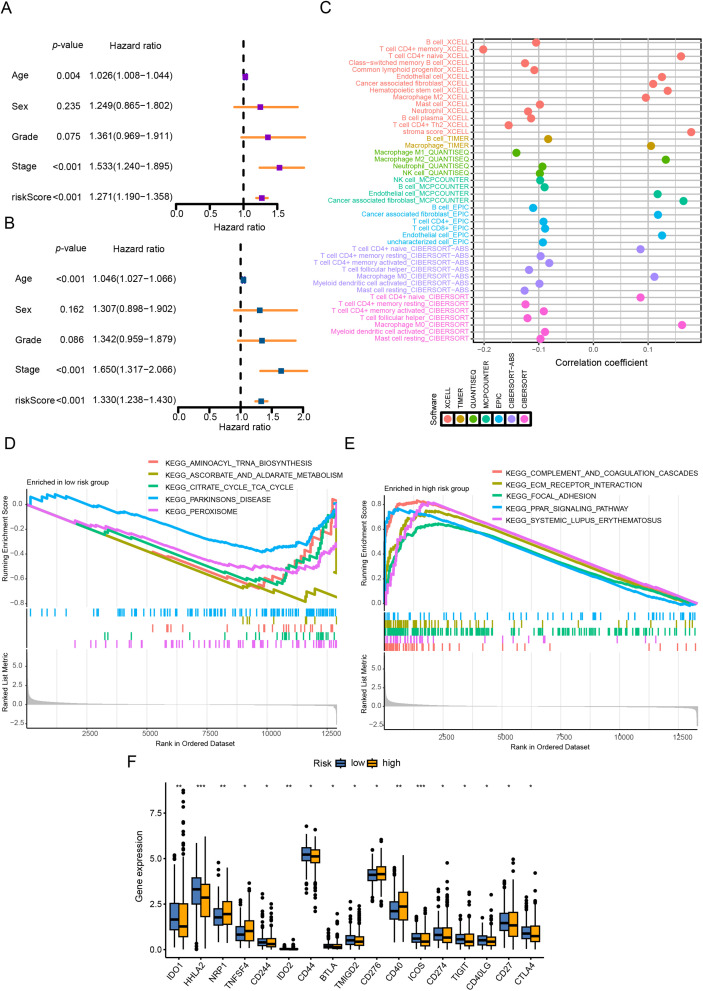
CRRG score as a prognostic indicator and immune landscape readout in CRC. (**A**) univariable Cox analysis of overall survival (OS) in the TCGA cohort; (**B**) multivariable Cox analysis of OS in the TCGA cohort; (**C**) correlation heatmap of 22 immune-cell fractions comparing high- vs. low-risk groups (TCGA); (**D, E**) GSEA results: high-risk tumors enriched for complement and coagulation cascades and ECM-receptor interaction; low-risk tumors enriched for immune function-related pathways; (**F**) immune checkpoint module scores contrasted between risk groups. *p < 0.05, **p < 0.01, ***p < 0.001

Univariable/multivariable Cox analyses in TCGA-CRC evaluated the CRRG score’s prognostic utility (Fig. 5A,B). Cox modeling (univariable and multivariable) identified the CRRG score as an independent prognostic variable (*p* < 0.05). Immune infiltration quantified by ssGSEA revealed that patients with lower CRRG scores exhibit increased abundance of B cells, resting/activated CD4^+^ memory T cells, T follicular helper cells, activated myeloid dendritic cells, resting mast cells, M1-polarized macrophages, and neutrophils (Fig. 5C). Enrichment of these subsets is generally indicative of an immune-inflamed TME that supports tumor elimination and favorable outcomes.

GSEA revealed distinct pathway activations between the risk groups: the high CRRG score group was predominantly enriched in immune- and stroma-associated pathways, such as complement and coagulation cascades and ECM-receptor interactions, suggesting enhanced immune and stromal remodeling activities. Conversely, the low CRRG score group showed enrichment in metabolic pathways, including aminoacyl-tRNA biosynthesis, ascorbate and aldarate metabolism, and the citrate (TCA) cycle, implying a metabolic reprogramming phenotype in these tumors (Fig. 5D,E).

In addition, assessment of immune checkpoint expression demonstrated elevated levels of immunosuppressive markers, such as CD276, CD40, ICOS, CD274, TIGIT, and CTLA4, in the high CRRG score group (Fig. 5F). This disparity indicates a differential immunotherapy response landscape, with the low CRRG score group likely to benefit more from immune checkpoint blockade. Taken together, the data demonstrate pronounced disparities in both immune infiltration and immune functional states between CRRG-defined groups, supporting their utility for tailoring immunotherapy.

### 4.5 Genomic Mutations and Drug Treatment Response Prediction across Different Groups

**Figure 6:**
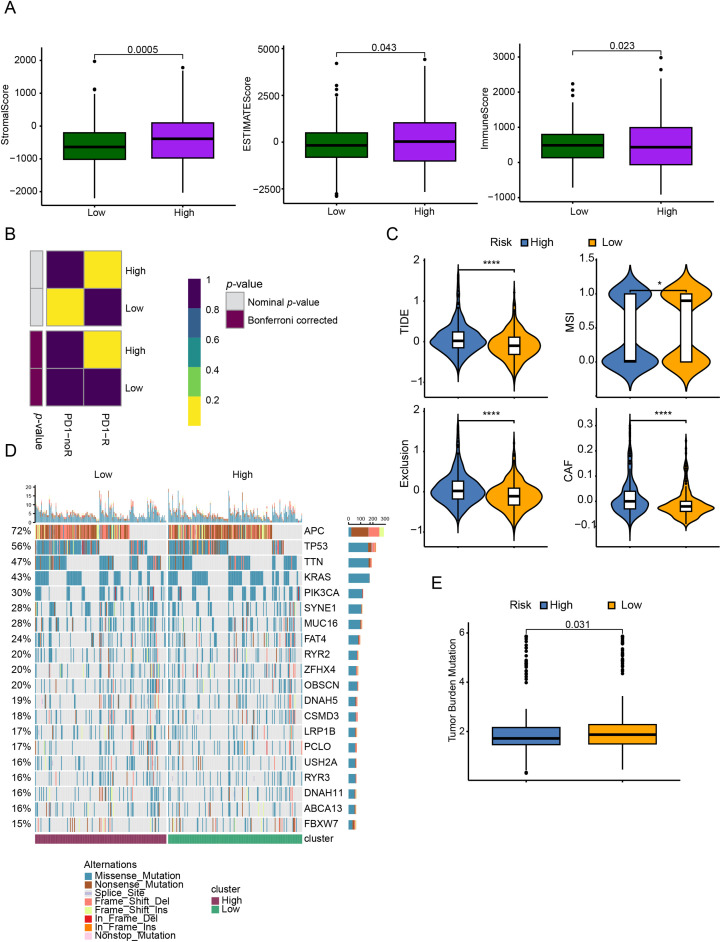
TME assessment and immune therapy response prediction between different risk groups. (**A**) ESTIMATE-derived scores (ESTIMATE score, ImmuneScore, StromalScore) compared between low- and high-risk groups. (**B**) Predicted responsiveness to anti–PD-1 therapy by TIDE and SubMap in low- vs. high-CRRGS tumors. (**C**) Distribution of composite TIDE scores between groups. (**D**) Waterfall (oncoplot) of the top 20 recurrently mutated genes in each group. (**E**) Differences in tumor mutational burden (TMB) between groups. *p < 0.05; ****p < 0.0001

Analysis using the Estimate algorithm demonstrated that patients with lower CRRG scores had elevated StromalScore, ESTIMATEScore, and ImmuneScore (Fig. 6A), reflecting a stronger degree of immune infiltration—a result that aligned with previous immune cell and cytokine analyses. The TIDE algorithm was subsequently employed to stratify patients into responders and non-responders based on gene expression profiles. A submap analysis further revealed statistically significant heterogeneity between response status and CRRG-defined risk groups, suggesting that the CRRG score has utility in predicting sensitivity to anti-PD1 immunotherapy (Fig. 6B). Consistently, the low-risk group exhibited significantly reduced TIDE scores, implying a higher probability of positive response to immunotherapy (Fig. 6C).

**Figure 7:**
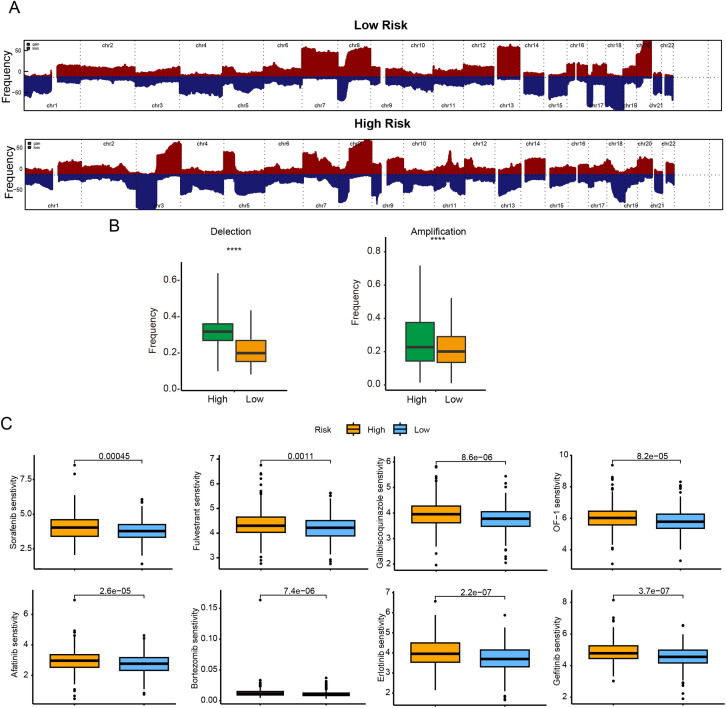
Genomic mutation profile and chemotherapy drug sensitivity comparison between different subgroups. (**A**) Frequencies of CNA in low- and high-risk groups; (**B**) box plots of copy-number gains (amplifications) and losses (deletions) by risk group; (**C**) predicted sensitivity to 8 commonly used chemotherapy drugs (Sorafenib, Afatinib, Fulvestrant, Bortezomib, Gallibiscoquinazole, Erlotinib, OF-1, and Gefitinib). IC_50_ values are significantly lower in the low-risk indicating higher chemotherapy sensitivity in the low-risk. ****p < 0.0001

In terms of genomic alterations, the top 20 most frequently mutated genes among CRC patients were visualized in a waterfall plot (Fig. 6D), with APC, TP53, TTN, KRAS, and PIK3CA being the predominant mutations. Notably, the low-risk cohort displayed a higher tumor mutational burden (TMB) (Fig. 6E). Because CNVs are key contributors to chromosomal instability, we compared CNV frequencies between risk groups and found that amplifications and deletions were markedly increased in the high-risk group (Fig. 7A,B).

To further corroborate the clinical relevance of the CRRG-based stratification, we assessed the sensitivity of both risk groups to commonly used chemotherapeutic agents by examining IC_50_ values. Lower IC_50_ values correspond to greater drug sensitivity and more favorable therapeutic effects. The low-risk subgroup showed enhanced sensitivity to Sorafenib, Afatinib, Fulvestrant, Bortezomib, Gallibiscoquinazole, Erlotinib, OF-1, and Gefitinib (Fig. 7C), indicating potential benefit from these therapies.

### 4.6 FAM84A Promotes CRC Malignant Progression

**Figure 8:**
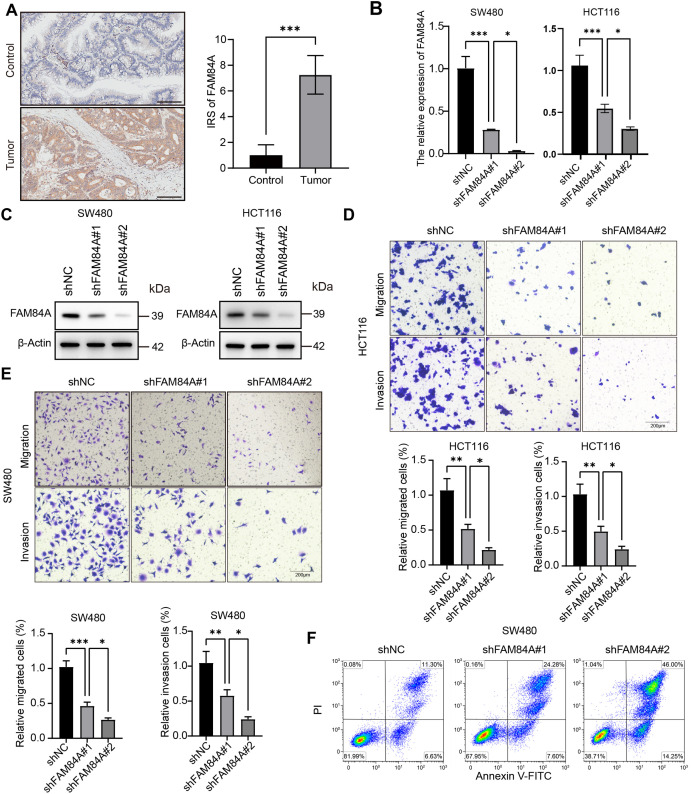

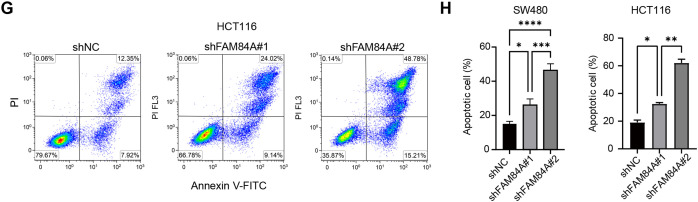
Functional validation of FAM84A as an oncogenic driver in CRC. (**A**) Immunohistochemistry (IHC) of FAM84A in CRC tissues, scale bar = 100 μm. (**B**) RT–qPCR quantification of FAM84A knockdown in SW480 and HCT116 cells transfected with shRNA-1/-2 vs. shNC. (**C**) Western blot analysis of FAM84A knockdown in SW480 and HCT116 with corresponding densitometry; (**D, E**) Transwell assays quantifying migration and invasion in SW480 and HCT116 cells (shNC vs shRNA-1/-2); (**F**–**H**) flow cytometric analysis of apoptosis in SW480 and HCT116 cells following FAM84A knockdown. *p < 0.05; **p < 0.01, ***p < 0.001, ****p < 0.0001

Among the six core genes incorporated into our prognostic signature, FAM84A was identified as the most critical contributor, achieving the highest feature importance ranking in the RSF algorithm. Despite its outstanding predictive value, the mechanistic role of FAM84A in CRC has not been thoroughly explored, making it an ideal target for experimental validation. In clinical CRC samples, FAM84A levels were markedly higher in tumors than in matched adjacent non-tumorous tissues (Fig. 8A). To assess its function, we knocked down FAM84A in two CRC cell lines, SW480 and HCT116, confirming successful silencing via PCR and Western blot analyses (Fig. 8B,C). Functional assays demonstrated that FAM84A knockdown markedly inhibited both the migratory and invasive abilities of CRC cells (Fig. 8D,E). Furthermore, flow cytometry analysis indicated that silencing FAM84A substantially increased apoptosis in CRC cells (Fig. 8F–H). Collectively, these *in vitro* findings suggest that FAM84A plays a pivotal role in promoting CRC cell malignancy.

**Figure 9:**
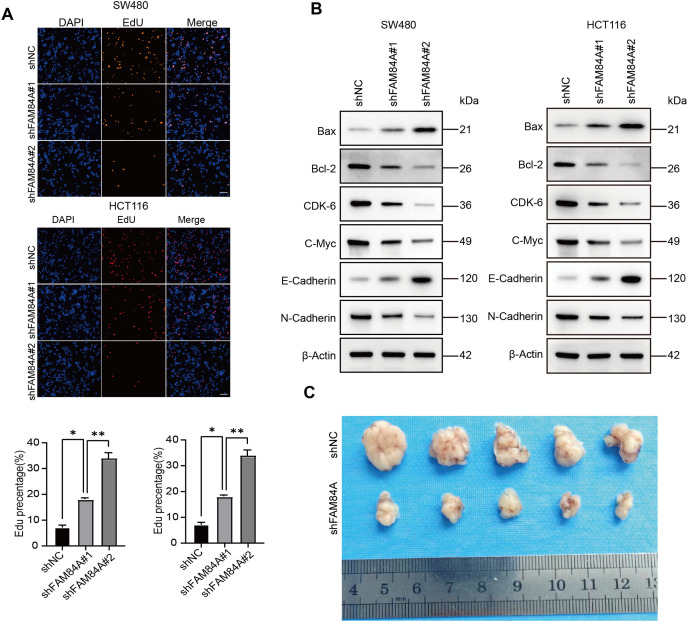

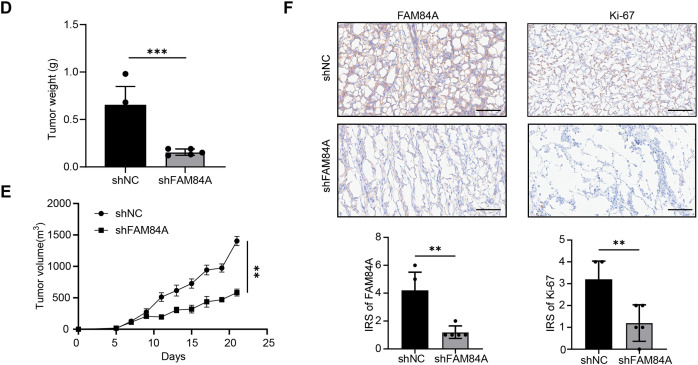
FFAM84A knockdown suppresses CRC progression *in vitro* and *in vivo*. (**A**) EdU assay of proliferative activity in SW480 and HCT116 cells transfected with control and FAM84A-targeting shRNAs (shNC vs. shRNA-1/-2); representative fields shown (×200; scale bar, 100 µm). (**B**) Western blot analysis of N-cadherin, E-cadherin, c-Myc, CDK6, Bcl-2, and Bax in SW480 and HCT116 cells following FAM84A knockdown (shRNA-1/-2 vs. shNC), with densitometric quantification. (**C**) Representative tumor photographs from each group at week 5. (**D**) Tumor weights at week 5. (**E**) Tumor volume growth curves over the treatment period. (**F**) Immunohistochemistry for FAM84A and Ki-67 in xenograft tumors (×200; scale bar, 200 µm). *p < 0.05; **p < 0.01, ***p < 0.001

Further functional characterization was performed using EdU incorporation assays, which showed a pronounced decrease in cell proliferation following FAM84A knockdown (Fig. 9A). Western blot results demonstrated that reduced FAM84A expression led to lower levels of N-cadherin, C-myc, CDK6, and Bcl-2, while levels of E-cadherin and pro-apoptotic Bax were increased, reflecting a shift toward a less aggressive, more apoptotic phenotype (Fig. 9B).To verify these effects *in vivo*, SW480 cells expressing either shNC or shFAM84A were subcutaneously injected into the right dorsal flank of C57BL/6 mice (n = 10; two groups, n = 5 per group). Tumor growth monitoring revealed that the shFAM84A group developed significantly smaller and lighter tumors than the shNC controls at 21 days post-inoculation (Fig. 9C–E). Immunohistochemical analysis further showed reduced expression of KI-67 and FAM84A in tumors from the shFAM84A group (Fig. 9F), confirming that FAM84A silencing effectively suppresses CRC progression *in vivo*.

## 5 Discussion

Recent studies reveal that the complement system functions not only in host defense but also as a key modulator of tumor-immune interactions [25–27]. Higher abundance of complement proteins C3 and C5 is linked to increased CRC risk and poorer prognosis [28], and have been implicated in treatment resistance and tumor progression [29]. Mechanistically, complement activation can promote immunosuppressive tumor-associated macrophage (TAM) polarization, angiogenesis, and metastasis through various pathways-including the NFAT1-C3a-C3aR [30] feedback loop and the JNK/STAT1 axis mediated by platelet-TAM interactions [31]. Moreover, the crosstalk between complement and coagulation systems in the TME further facilitates tumor invasion [32] and dissemination [33]. In our study, complement and coagulation cascade-related genes effectively stratified CRC patient prognosis and immune infiltration, underscoring their central regulatory role in the inflammatory and immune landscape of tumors. In this study, our model focuses on complement and coagulation cascade-related genes, which in bioinformatics analyses significantly stratified the survival and immune infiltration status of CRC patients. These findings suggest that these genes may regulate the inflammatory and immune balance within the tumor microenvironment.

Differences in the C1 and C2 clusters appear to shape CRC aggressiveness and progression, thereby influencing patient outcomes. Functional enrichment of differentially expressed genes highlighted strong signals in cell-cycle programs and ECM–receptor interaction pathways, offering a mechanistic rationale for the prognostic gap between clusters. We then built a prognostic biomarker model using ten algorithms combined into 101 methodological ensembles; among these, the random survival forest (RSF) delivered the best performance and was selected for downstream analyses. TCGA served as the training cohort and GSE39582 as an external validation cohort, with patients stratified into high- and low-risk groups by the derived risk score to verify model robustness. Beyond mere separation by gene signatures, our framework incorporates complement-cascade features into prediction, enabling more individualized risk assessment and informing therapeutic decision-making.

Ultimately, six genes were identified for the construction of the prognostic model: GPX3, FAM84A, IGFBP3, PTPRD, IDO1, and CYP2W1. GPX3, whose promoter methylation is found in ~30% of CRC cases, suppresses oxidative stress and its loss correlates with enhanced platinum sensitivity, making it a predictive marker for chemotherapy [34,35]. Promoter methylation-mediated silencing of IGFBP3 characterizes CRC and is tightly linked to lymph-node involvement and poor prognosis in stage II–III cases [36]. PTPRD, a receptor-type tyrosine phosphatase, is frequently inactivated in CRC; its loss is associated with increased invasion and worse prognosis [37]. IDO1, highly expressed at CRC invasive fronts, catalyzes tryptophan catabolism to suppress T cell activity and is an marker for poorer overall and metastasis-free survival [38]. CYP2W1, selectively expressed in ~30–36% of colon tumors, is an independent prognostic factor in stage II-III disease, likely via activation of procarcinogens and altered drug metabolism [39]. Initially identified via cDNA microarray as being highly upregulated in CRC, but not in normal tissues except testis-FAM84A localized to cell membrane edges and its overexpression increased cell motility in NIH3T3 fibroblasts; serine-38 phosphorylation was linked to morphological changes and enhanced migration [40]. Moreover, FAM84A overexpression in papillary thyroid carcinoma-promoting proliferation, EMT, invasion, and Wnt/β-catenin pathway activation-partially regulated by miR-874-3p [41].

By integrating genes with diverse functions-including redox balance, oncogenic signaling, immune regulation, and metabolic adaptation-our model offers a more comprehensive reflection of CRC biology than traditional biomarkers. Notably, the inclusion and functional validation of FAM84A provide new mechanistic insight and enhance the model’s predictive accuracy, supporting its value for individualized risk assessment and clinical decision-making in CRC. The study analyzed the role of the prognostic model gene FAM84A in CRC. FAM84A shares an amino acid sequence with human collagen types X and α1 and is considered a novel collagen protein [42]. We confirmed that FAM84A is markedly upregulated in CRC tissues and cell lines. Using EdU and Transwell assays, we further showed that silencing FAM84A suppresses CRC cell proliferation, migration, and invasion. WB analysis revealed that FAM84A knockdown led to a decrease in N-cadherin, C-myc, CDK6, and Bcl-2 levels, while E-cadherin and Bax expression increased, indicating that FAM84A plays a critical regulatory role in tumor cell growth, metastasis, and apoptosis. The downregulation of N-cadherin suggests that FAM84A may influence the epithelial-mesenchymal transition (EMT) process [43], reducing the migration and invasion abilities of tumor cells. The decrease in C-myc and CDK6 is typically associated with cell cycle arrest and proliferation inhibition [44,45] highlighting the role of FAM84A in regulating tumor cell proliferation. The reduction in Bcl-2, an anti-apoptotic protein [46], indicates that knockdown of FAM84A may promote cell apoptosis. Concurrently, the upregulation of E-cadherin reflects enhanced cell-cell adhesion, likely due to the suppression of EMT [47]. The increase in Bax further supports enhanced apoptosis, as Bax is a pro-apoptotic protein [48], suggesting that FAM84A knockdown may promote tumor cell death through this pathway. Highly FAM84A-expressing aggressive tumors frequently exploit inflammatory signaling (including certain complement pathways) to achieve immune evasion. However, it is regrettable that the relationship between complement and FAM84A requires further investigation to determine whether they directly interact. Within the model, this association is represented as an independent oncogenic axis. In sum, our findings nominate FAM84A as a promising biomarker for CRC prognosis and disease progression and highlight its potential as a therapeutic target to inform clinical decision-making.

In CRC, the TME exerts a major impact, with differential immune-cell infiltration shaping prognosis. Consistent with this, patients classified as low risk exhibit an immune-inflamed microenvironment, displayed enriched infiltration by multiple immune lineages, including B cells, CD4^+^ memory T cells, Tfh, activated myeloid dendritic cells, and M1 macrophages-populations generally linked to antitumor immunity.B cells contribute to sustained immune responses by producing antibodies, promoting antigen presentation, and establishing immune memory. CD4^+^ memory T cells and Tfh cells are critical for maintaining and amplifying T cell immune responses, particularly playing an essential role in immune surveillance within the tumor microenvironment [49]. M1 macrophages contribute by producing pro-inflammatory cytokines to eliminate tumor cells, thus, the accumulation of these immune cells within the tumor often signals a healthier immune environment, correlating with a better prognosis for the patient [50]. The presence of resting mast cells and neutrophils suggests that this group may possess strong immune defense capabilities, aiding in the clearance of tumor cells or preventing tumor spread. Although the high-risk group displays higher immune checkpoint expression, recent studies have indicated that patients with tumors elevated PD-1, PD-L1, or CTLA-4 expression are more likely to benefit from immune checkpoint blockade, as these markers often reflect a suppressed but immunologically active tumor microenvironment [51,52]. The presence of these immune populations likely contributes to enhanced immune surveillance and sustained anti-tumor activity, which may, in turn, improve response to immunotherapies.

Among biomarkers relevant to immunotherapy, TMB is considered a major contributor to treatment outcome. Tumors with higher TMB typically present more neoantigens, which can activate stronger T cell immune responses [53]. Thus, the low CRRG score group may have a higher TMB, but High immune-cell infiltration sustains surveillance and response; alongside relatively high TMB, it may boost neoantigen visibility and improve immune recognition and killing of tumor cells. Moreover, patients with lower TMB may exhibit stronger immune cell infiltration, which could potentially improve the effectiveness of immunotherapy, particularly in cases where the immune system overcomes tumor immune escape mechanisms through the synergistic action of immune cells. To further assess this, An additional assessment of immunotherapy sensitivity revealed that TIDE scores were decreased in low-risk patients relative to high-risk counterparts, supporting enhanced responsiveness in the low-risk subset. From a genomic standpoint, these tumors were more stable, with diminished CNV burden—specifically, fewer amplification and deletion events [54]. Amplifications and deletions often result in the overexpression or loss of hub genes, which can affect the efficacy of drug targets and subsequently lead to drug resistance. For example, gene amplification may enable tumor cells to evade the inhibitory effects of drugs, while gene depletion can result in the loss of suppressive genes, conferring greater drug resistance to the tumor cells [55]. In the low-risk group, the relatively stable genomic structure and the reduced occurrence of amplifications and deletions suggest that these tumors are less resistant to drugs. Consequently, the drugs can more precisely target molecular pathways and inhibit tumor cell growth. In the context of better prognosis in the low-risk group in TCGA cohorts, the enhanced sensitivity to various drugs can typically be explained by the genomic features and expression patterns of drug targets. Such genomic features are increasingly recognized as determinants of drug sensitivity, since tumors with fewer amplifications or deletions tend to have more stable targets and reduced resistance, thereby benefiting more from targeted therapies. For drugs like Sorafenib, Afatinib, Fulvestrant, Bortezomib, Gallibiscoquinazole, Erlotinib, OF-1, and Gefitinib, their higher sensitivity is often associated with specific molecular characteristics and genomic stability within the low-risk group. Tumor cells in this group typically exhibit distinct gene expression profiles that enhance the efficacy of these drugs. For instance, Sorafenib and Erlotinib are multi-target tyrosine kinase inhibitors whose mechanisms of action involve inhibiting signaling pathways such as VEGF and EGFR pathways to block tumor angiogenesis and cell proliferation [56,57]. In the low-risk group, due to the relatively low mutational burden and specific abnormalities in certain signaling pathways, the targets of these drugs are more exposed and susceptible to inhibition, potentially increasing the therapeutic effectiveness. Afatinib and Gefitinib, as EGFR-targeted therapies [58,59], are closely related to EGFR mutations in tumor cells. The mutation patterns of EGFR in the low-risk group may contribute to the heightened sensitivity to these drugs. The presence of specific EGFR mutations in this population likely facilitates the effective targeting of these therapies, enhancing their clinical efficacy.

However, there are several limitations in this study. Firstly, although the study constructed a prognostic risk model using machine learning, the model still relies on gene expression data from publicly available databases, which may introduce sample heterogeneity and data bias. Secondly, although this study revealed the potential role of the complement cascade in the TME of CRC, further experimental validation is lacking, and more prospective preclinical studies are needed. Additional clinical samples will be collected for validation, and the research will be extended to other cancers. Lastly, The precise mechanisms by which FAM84A acts remain incompletely defined. Nevertheless, our data show that FAM84A silencing suppresses CRC cell proliferation, migration, and invasion in both *in vitro* and settings, the specific signaling pathways and whether FAM84A influences tumor progression through complement-related pathways require further investigation. Although our findings link FAM84A closely to the immune microenvironment and CRC prognosis, its specific role in immune evasion remains to be explored in more depth through basic research. In summary, this study contextualizes the complement system’s multifaceted involvement in CRC prognosis and therapy, and highlights the translational promise of integrated biomarker-driven models for guiding personalized treatment.

## Data Availability

All data generated or analyzed during this study are included in this published article.
